# A revision of the geographical distributions of the shrews *Crocidura tanakae* and *C. attenuata* based on genetic species identification in the mainland of China

**DOI:** 10.3897/zookeys.869.33858

**Published:** 2019-08-05

**Authors:** Yaoyao Li, Haotian Li, Masaharu Motokawa, Yi Wu, Masashi Harada, Huimei Sun, Xinmin Mo, Jing Wang, Yuchun Li

**Affiliations:** 1 Marine College, Shandong University (Weihai), Weihai 264209, China Shandong University Weihai China; 2 The Kyoto University Museum, Kyoto University, Kyoto 606-8501, Japan Kyoto University Kyoto Japan; 3 School of Life Sciences, Guangzhou University, Guangzhou 510006, China Guangzhou University Guangzhou China; 4 Laboratory Animal Center, Osaka City University, Osaka 545-8585, Japan Osaka City University Osaka Japan

**Keywords:** *Crocidura attenuata*, *Crocidura tanakae*, geographical distribution, mainland of China, Taiwan Island

## Abstract

The Taiwanese gray shrew (*Crocidura tanakae*) and Asian gray shrew (*C. attenuata*) are so similar in size and morphology that the taxonomic status of the former has changed several times since its description; *C. tanakae* has also been regarded as an endemic species of Taiwan Island. In recent years, molecular identification has led to several reports of *C. tanakae* being distributed in the mainland of China. In this study, we determine the geographical distribution of *C. attenuata* and *C. tanakae* based on more than one hundred specimens collected during 2000 to 2018 over a wide area covering the traditional ranges of the two species in the mainland of China, and show a substantial revision of their distributions. Among 110 individuals, 33 *C. attenuata* and 77 *C. tanakae* were identified by *Cytb* gene and morphologies. Our results show, (1) *C. attenuata* and *C. tanakae* are distributed sympatrically in the mainland of China; (2) contrary to the previous reports, the distribution range of *C. attenuata* is restricted and much smaller than that of *C. tanakae* in the mainland of China; (3) Hainan Island, like Taiwan Island, is inhabited by *C. tanakae* only according to the present data.

## Introduction

The Taiwanese gray shrew (*Crocidura tanakae* Kuroda, 1938) and Asian gray shrew (*C. attenuata* Milne Edwards, 1872) are distinct species with very similar morphological characters and measurements, such that the taxonomic status of *C. tanakae* has been changed several times by taxonomists. *Crocidura tanakae* was originally described from Taiwan as a new species by Kuroda (1938); however, because it could not be distinguished from *C. attenuata* in morphological characters and measurements, *C. tanakae* was thereafter regarded as a synonym or subspecies, *C. a. tanakae* by many authors (Ellerman and Morrison-Scott 1951; Jameson and Jones 1977; Corbet and Hill 1992; Hutterer 1993; Fang et al. 1997;). Motokawa et al. (2001) recognized the distinct taxonomic position of *C. tanakae* by chromosomal data, and regarded it as the endemic species of Taiwan Island.

**Figure 1.**
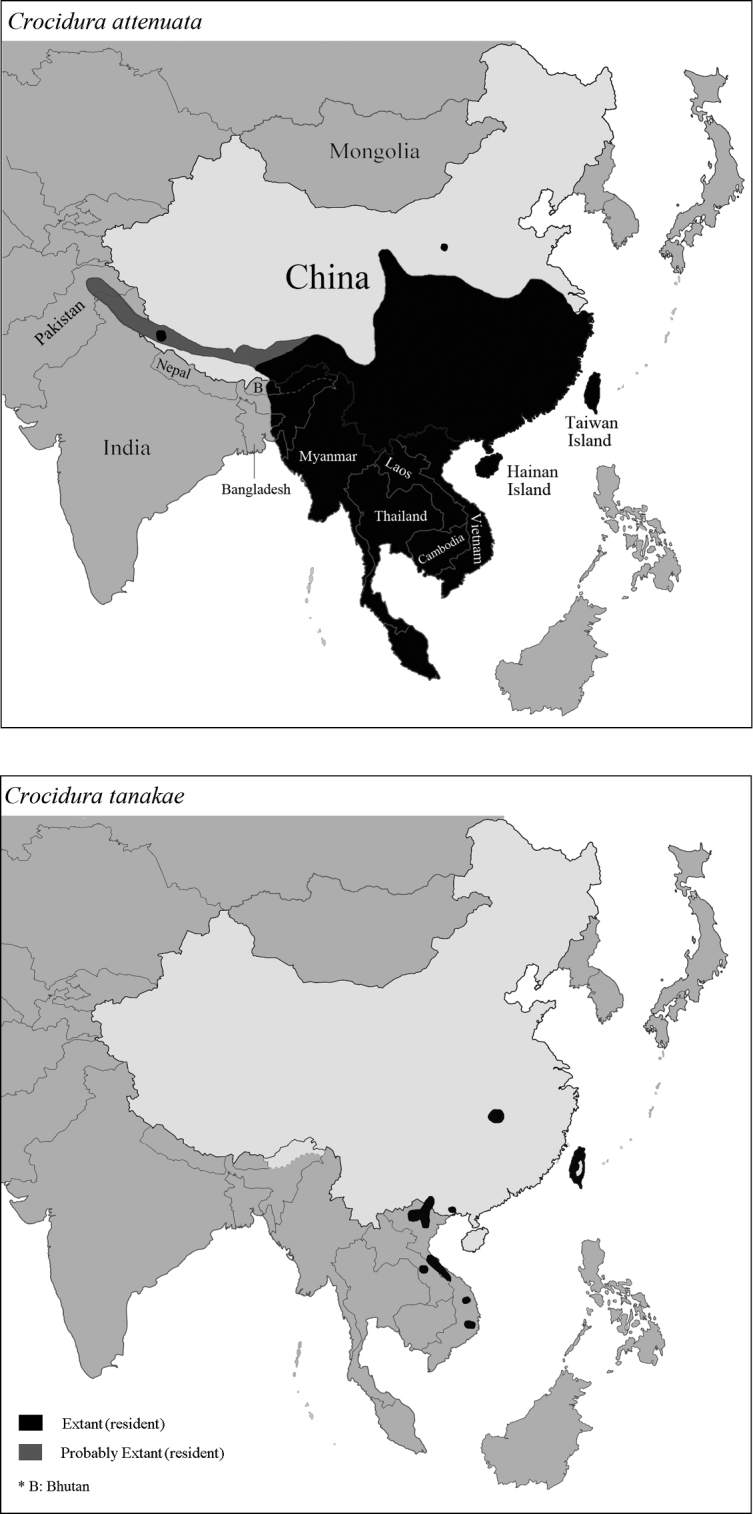
Distributions of *Crocidura attenuata* and *C. tanakae* by the IUCN.

In recent years, the application of molecular identification techniques led to reports of *C. tanakae* populating the mainland of China. Esselstyn et al. (2009) and Esselstyn and Oliveros (2010) genetically identified specimens collected in Vietnam and the Hunan and Guizhou Provinces of China and found most of their specimens belonged to *C. tanakae*; only a few were attributed to *C. attenuata*. Bannikova et al. (2011) and Abramov et al. (2012) reported that *C. tanakae* was also found in Vietnam and Laos, and it was a wide-spread species in Vietnam, whereas *C. attenuata* inhabited only the north and east of the Red River; Chinese scientists recently reported *C. tanakae* was collected from the mainland of China including Mount Emei of Sichuan Province, Mount Fanjing of Guizhou Province, Pingbian and Funing of Yunnan Province and Xingshan of Hubei Province (Cheng et al. 2017; Chen et al. 2018; Lei et al. 2019). However, these reports only provided the data for several distribution areas and were not sufficient to generalise the overall distributions of the two species in the mainland of China. The current IUCN distribution maps of *C. attenuata* and *C. tanakae* presented in Figure 1 are revised by this study.

We accumulated more than one hundred specimens from 19 areas of *C. attenuata* and *C. tanakae* in our field surveys in the mainland of China from 2000 to 2018, which expands the previous distributions from the aforementioned reports from a few localities. A re-evaluation of geographical distributions of the two species is important to a range of studies and practical needs, such as zoogeography, geophylogeny, agriculture animal management, health and epidemic prevention. Here we report the wide geographical distributions of *C. attenuata* and *C. tanakae* in the mainland of China.

## Materials and methods

### Samples and DNA sequencing

A total of 110 specimens of the *C. attenuata* complex were collected from 11 provinces in the mainland of China including Anhui (2 specimens), Fujian (4), Guangdong (23), Guangxi (4), Hubei (4), Hunan (3), Jiangxi (13), Sichuan (Chongqing is included) (30), Yunnan (1) and Zhejiang (23) as well as the Hainan Island (2) using Sherman live cages during 18 years from Aug. 2000 to Sep. 2018. The geographical position (longitude and latitude) of each specimen was recorded by GPS (Suppl. material 1, Table S1). All specimens including pelt, carcass and skulls were kept in Shandong University (Weihai) and Guangzhou University.

DNA was extracted from muscle samples using the Ezup Column Animal Genomic DNA Purification Kit (Sangon Biotech Co., Ltd., Shanghai, China). The complete mitochondrial cytochrome *b* gene (*Cytb*, 1140 bp) was amplified by PCR with the primers as in Irwin et al. (1991). Primers Nivicob1 (5’-TGTCATTATTTCTACACAGCACTTA-3’) and Nivicob2 (5’-TTTGGGTGTTGATGGTGGG-3’) were used for amplification of the whole *Cytb* gene. PCR reactions were 25 μL, containing 0.25 μM primers, 2×EasyTap PCR Supermix 12.5 μL, and approximately 15 ng DNA template. The thermocycling protocol was as follows: an initial denaturation of 5 min at 95 °C; 32 cycles of 95 °C for 30 s, annealing temperature (Tm) for 30 s, 72 °C for 1 min; a final extension of 10 min at 72 °C. PCR products were directly sequenced by Sanger sequencing technique.

### Phylogenetic analyses

*Cytb* gene sequences were aligned using BioEdit v.7.2.5 (Hall 1999). Each specimen was molecularly identified for species by blasting on GenBank and confirmed by ML (maximum likelihood) phylogenetic tree construction in MEGA 5 (Tamura et al. 2011) based on TN93+G model. We used the Akaike Information Criterion (AIC) in jModeltest1.0 (Posada 2008) to select the best-fit model of sequence evolution for the locus alignment. The bootstraps were obtained using a rapid bootstrapping algorithm with 1000 replicates. We calculated the genetic distance of Kimura-2-parameter (K2P) of *Cytb* between the two species.

We also included *Cytb* sequence data from several earlier studies (Ohdachi et al. 2004, 2006; Bannikova et al. 2006, 2009, 2011; Jenkins et al. 2009, 2013; Esselstyn and Oliveros, 2010; Abramov et al. 2012; Chen et al. 2016) to place the shrews from type locality and Vietnam into a phylogenetic context, the sequence information was showed in Suppl. material 1, Table S2. *Suncus murinus* was selected as outgroup (Suppl. material 1, Table S2). GenBank accession numbers for the original sequences used in this study were MK765682-MK765791 (Suppl. material 1, Table S1).

### Morphological analyses

In order to attribute these genetic lineages to taxonomically correct species names, we photographed the dorsal, ventral, lateral of skull and lateral view of the mandible of *C. attenuata* from type locality – Baoxing (Moupin), Sichuan – and also photographed the corresponding teeth, and marked the characteristic features on the pictures for this species. We repeated the same procedure with the only sample of *C. tanakae* from the same locality (Baoxing) for interspecific comparisons.

We conducted a morphological investigation of the specimens sampled to identify the two species by determining three external measurements: total body length (TBL), head and body length (HBL), ear length (EL); and 10 skull measurements: greatest length of skull (GLS), cranial base length (GBL), median palatal length (MPL), length of teeth row (LUTR), greatest palatal breadth (GPB), breadth of occipital condyles (BOC), greatest breath of braincase (BBC), interorbital breadth (IOB), height of the braincase (HB), length of mandible (LM) according to Yang et al. (2005, 2007) and Jenkins et al. (2009).The measurements of the skull indices were performed with a digital vernier caliper (0.01 mm). Juveniles and sub-adults were excluded from the analysis according to the complete fusion of cranial sutures (Motokawa et al. 1997, 2003), and by making a histogram of the HBL as an indicator for age identification of small mammals (Li et al. 1989, 1990; Yang 1990).

We calculated the mean and standard deviation of external and skull morphological indices. The pairwise differences between the two species were tested by independent sample *t*-tests or Mann-Whitney *U* tests according to results of the Kolmogorov-Smirnov test for their normality of distribution. Principal component analysis (PCA) was used to test the general appropriateness of the groupings supplied by assessment of overall variation in the skull characters. These analyses were performed using SPSS Statistics 24.0 (SPSS, Chicago, IL, USA).

## Results

**Figure 2.**
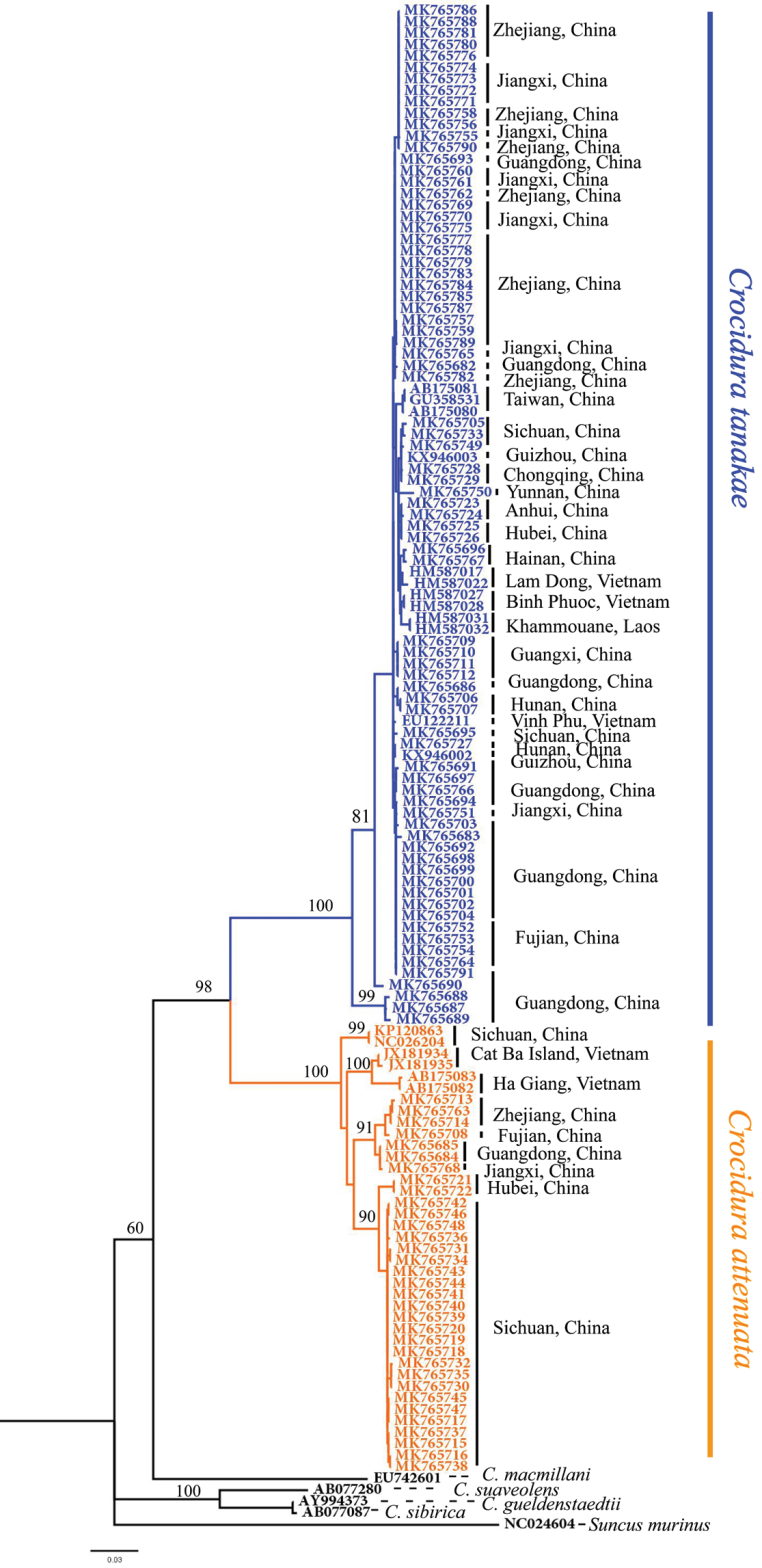
ML tree based on *Cytb* of *Crocidura* genus. Numbers above the branches represent bootstrap support (BS). The blue clade represents *C. tanakae* and orange clade represents *C. attenuata*.

**Figure 3.**
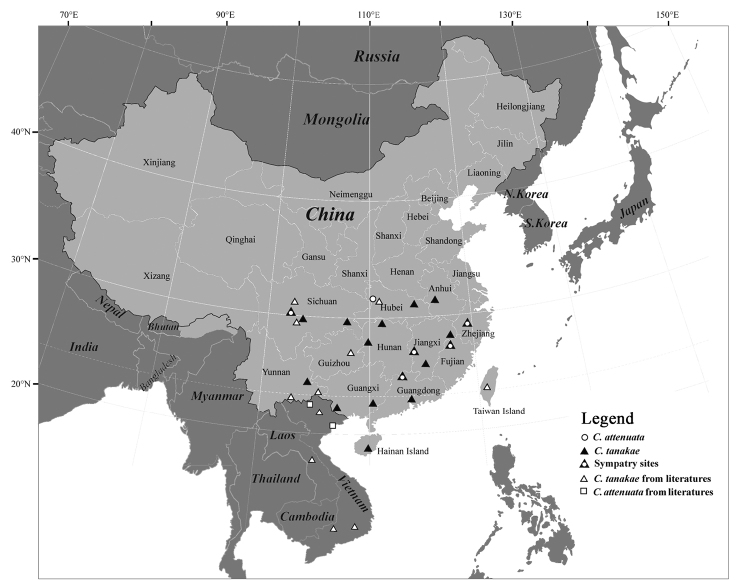
Distributions of *Crocidura attenuata* and *C. tanakae* in the mainland of China，Laos and Vietnam. Black and white triangles represent the sampled sites of *C. tanakae* first presented in this study and in previous studies, respectively. White circles and squares represent the sampled sites of *C. attenuata* first presented in this study and in previous studies. Black triangles and white circles overlapped indicate sympatry sites.

We obtained 1140 bp of mitochondrial DNA sequences from 110 individuals in this study. The ML tree indicated that the specimens we collected were divided into two lineages, one was clustered with the *C. attenuata* download from GenBank which was distributed in its type locality, i.e., Baoxing of Sichuan Province, and the other was clustered with the *C. tanakae* download from GenBank which was exclusively distributed in its type locality, i.e., Taiwan Island (Fig. 2). K2P distance of *Cytb* between these two lineages was 12.3%. Together with the results of blasting on GenBank, a total of 33 specimens of *C. attenuata* lineage and 77 specimens of *C. tanakae* lineage collected in this study were genetically identified by *Cytb*, and their distribution localities plotted in Figure 3. Also, the distribution localities of *C. tanakae* recently reported in the mainland of China were added to the figure.

**Figure 4.**
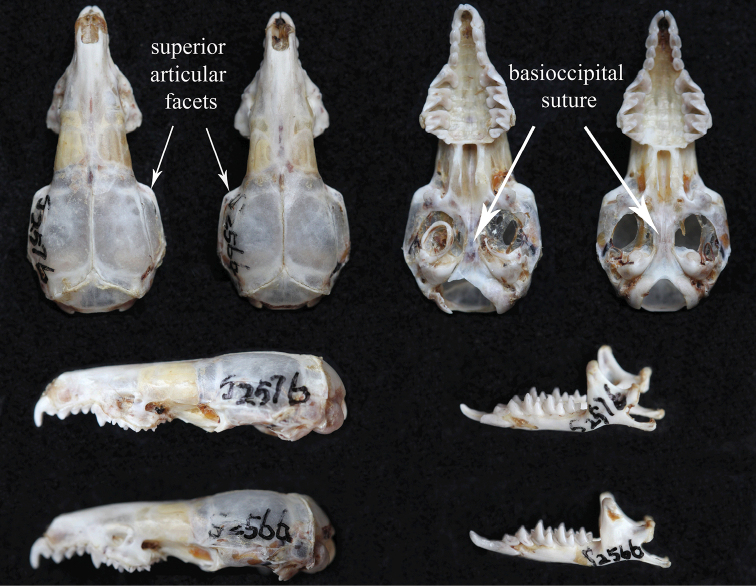
Comparison of crania of *Crocidura attenuata* (S2576) and *Crocidura tanakae* (S2566) from Baoxing, Sichuan. Top row from left to right: dorsal views of the skulls of *C. attenuata* and *C. tanakae* (S2576 andS2566), ventral views of the skulls in the same order. Lower row: lateral view of skulls and mandibles from top to bottom of *C. attenuata* and *C. tanakae* (S2576 and S2566).

**Figure 5.**
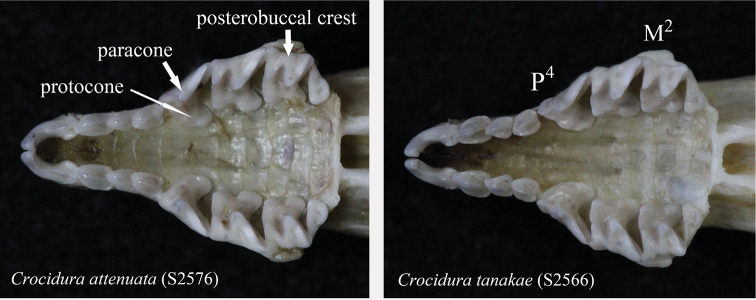
Comparison of teeth of *Crocidura attenuata* (left: S2576) and *Crocidura tanakae* (right: S2566) from Baoxing, Sichuan.

By investigating our samples of *C. attenuata* lineage from Baoxing, Sichuan, we found some morphological features correlated with the holotype: the superior articular facets are more angular in dorsal view and the basioccipital region is narrow and ridged particularly anterior to the position of the basioccipital suture in *C. attenuata* (Fig. 4). On the upper premolar (P^4^) the protocone is variably positioned relative to the paracone; the posterolingual border of the tooth is not so rounded; and the posterior border of the tooth is deeply concave. The posterobuccal crest of the paracone of the second upper molar (M^2^) forms a smooth W-shaped loph in unworn dentition (Fig. 5).

**Figure 6.**
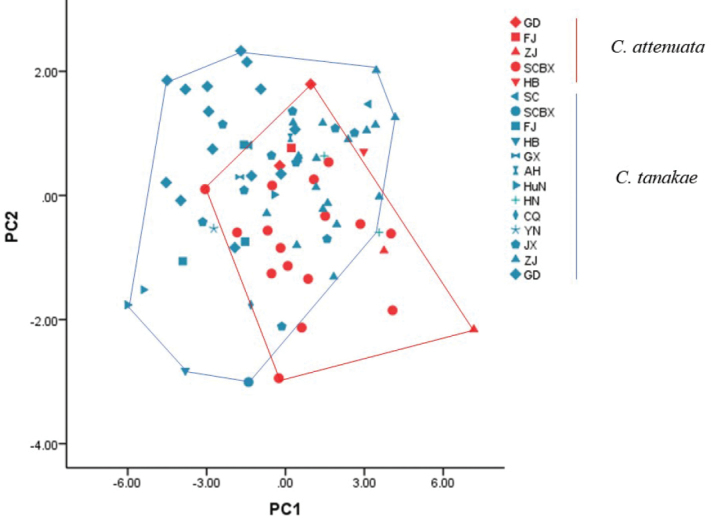
Scatter plot of *C. attenuata* (red) and *C. tanakae* (blue) sample distribution over PC1 and PC2 axes constructed based on external and skull morphological variables. Different symbols represent different populations. GD: Guangdong, FJ: Fujian, ZJ: Zhejiang, SCBX: Baoxing, Sichuan, HB: Hubei, SC: Sichuan, GX: Guangxi, AH: Anhui, HuN: Hunan, HN: Hainan, CQ: Chongqing, YN: Yunnan, JX: Jiangxi

A total of 90 adult individuals were screened by age identification including 26 *C. attenuata* and 64 *C. tanakae*. The external and two skull measurements (BOC and GPB) were judged as a non-normal distribution by the Kolmogorov-Smirnov test (*P*<0.05), so we used the Mann-Whitney *U* Test for interspecific comparisons; for the others with normal distribution (*P*>0.05) the parametric independent sample *t*-test was used (Suppl. material 1, Table S3). Descriptive statistics for external and craniodental measurements of the two species and literature measurements (including holotype) are given in Table 1; they were basically consistent with the variation range and limits recorded in the literature except for IOB. *Crocidura attenuata* was a little larger than *C. tanakae* in GBL, MPL and BBC. Although there existed significant differences (*P*<0.05) in some morphological indices between the two species (Table 2), their range of measurements greatly overlapped. In the PCA made on external and skull measurements, three principal components were extracted and captured 70.07% of the total variation. Five indices, GBL, GLS, LUTR, BBC and LM, were the top five with the highest correlations with the first axis (PC1, Table 3). The sample distributions over the scatter plot in coordinate area constructed by first two principal component axes showed a great overlap between the two species in external and skull indices (Fig. 6), indicating that morphological indices cannot accurately identify the two species.

**Table 1.** External and cranial measurements of *C. attenuata* and *C. tanakae* (in millimeters). Mean ± standard deviation (SD), and range, with number of specimens in parentheses are presented for characters available.

**Morphological indices**	***Crocidura attenuata***		***Crocidura tanakae***			
	This study	Jenkins et al. 2009 (including holotype)	This study	Kuroda 1938 (Holotype)	Fang et al. 1997	Motokawa et al. 2001
Total Body Length (TBL)	133.92±6.22	–	132.95±6.84	–	–	–
	120.00–144.00 (26)	–	115.00–148.00 (64)	–	–	–
Head and Body Length (HBL)	77.96±3.54	71.40±6.64	78.90±5.42	66.00	77.90±3.60	73.36±6.67
	71.00–87.00 (26)	63.00–82.00 (10)	70.00–95.00 (64)	–	69.70–86.00	64.5–84.5
Ear Length (EL)	8.32±1.05	–	8.79±1.00	–	8.96±0.39	9.05±0.91
	6.05–10.16 (26)	10.00 (2)	5.97–11.06 (63)	–	8.10–9.80	7.9–10.2
Greatest Length of Skull (GLS)	20.99±0.59	20.55±0.66	20.54±0.61	20.00	20.84±0.42	20.85±0.41
	20.12–22.36 (23)	19.60–21.70 (9)	19.23–21.69 (61)	–	20.03–21.82	19.94–21.31
Cranial base Length (GBL)	20.91±0.57	–	20.39±0.61	–	19.88±0.46	–
	20.04–22.26 (24)	–	18.85–21.68 (61)	–	19.06–20.73	–
Median palatal Length (MPL)	9.07±0.41	–	8.89±0.31	–	–	–
	8.35–10.13 (26)	–	8.14–9.61 (63)	–	–	–
Length of tooth row (LUTR)	9.36±0.26	8.72±0.38	9.16±0.34	9.00	9.29±0.16	9.33±0.19
	8.97–9.96 (26)	8.20–9.50 (11)	8.19–9.91 (63)	–	8.73–9.62	8.87–9.54
Greatest palatal breadth (GPB)	6.25±0.16	6.09±0.22	6.32±0.31	–	6.33±0.16	6.41±0.13
	5.96–6.55 (26)	5.80–6.50 (11)	5.65–6.97 (64)	–	5.98–6.68	6.20–6.58
Breadth of Occipital Condyles (BOC)	5.20±0.19	–	5.13±0.23	–	–	–
	4.78–5.55 (22)	–	4.48–5.64 (60)	–	–	–
Greatest Breath of Braincase (BBC)	9.59±0.27	9.17±0.24	9.26±0.32	–	9.23±0.18	9.22±0.22
	8.98–10.25 (26)	8.70–9.40 (8)	8.57–10.02 (64)	–	8.00–9.66	8.87–9.50
Interorbital Breadth (IOB)	3.97±0.23	4.43±0.18	3.79±0.17	4.50	4.50±0.10	4.62±0.10
	3.61–4.46 (26)	4.10–4.70 (9)	3.43–4.23 (64)	–	4.29–4.68	4.47–4.74
Height of the Braincase (HB)	5.13±0.14	4.91±0.10	5.01±0.15	–	–	–
	4.80–5.42 (26)	4.80–5.10 (8)	4.65–5.35 (64)	–	–	–
Length of mandible (LM)	10.01±0.28	12.76±0.62	9.85±0.33	–	8.38±0.20	–
	9.34–10.67 (26)	11.7–13.9 (11)	9.06–10.52 (64)	–	7.88–8.91	–

**Table 2.** Morphological comparisons and significant difference between *C. attenuata* and *C. tanakae* in this study. Values in bold show significant differences.

**Morphological indices**	**T test/Mann-Whitney U Test**					
	**F**	**Sig.**	**t**	**Df (N)**	**Z**	**P**
Total Body Length (TBL)				(90)	-0.687	0.492
Head and Body Length (HBL)				(90)	-0.478	0.633
Ear Length (EL)				(89)	-2.08	**0.038**
Greatest Length of Skull (GLS)	0.149	0.701	-3.045	82		**0.003**
Cranial base Length (GBL)	0.133	0.717	-3.541	83		**0.001**
Median palatal Length (MPL)	1.214	0.274	-2.263	87		**0.02**6
Length of teeth row (LUTR)	1.174	0.282	-2.615	87		**0.011**
Greatest palatal breadth (GPB)				(90)	-1.077	0.281
Breadth of Occipital Condyles (BOC)				(82)	-1.256	0.209
Greatest Breath of Braincase (BBC)	1.416	0.237	-4.666	88		**<0.001**
Interorbital Breadth (IOB)	2.492	0.118	-4.143	88		**<0.001**
Height of the Braincase (HB)	1.131	0.29	-3.61	88		**0.001**
Length of mandible (LM)	2.766	0.1	2.195	88		**0.031**

**Table 3.** Principal component loadings as performed by analyses of 13 morphological measurements of *C. attenuata* and *C. tanakae*.

**Variable**	**Component**		
	**PC1**	**PC2**	**PC3**
Total Body Length (TBL)	0.691	0.550	0.065
Head and Body Length (HBL)	0.435	0.607	0.458
Ear Length (EL)	0.101	0.706	-0.314
Greatest Length of Skull (GLS)	0.948	-0.088	-0.062
Cranial base Length (GBL)	0.966	-0.096	-0.055
Median palatal Length (MPL)	0.815	0.041	-0.372
Length of teeth row (LUTR)	0.867	-0.139	0.017
Greatest palatal breadth (GPB)	0.566	0.138	0.458
Breadth of Occipital Condyles (BOC)	0.545	-0.358	0.561
Greatest Breath of Braincase (BBC)	0.837	-0.140	-0.022
Interorbital Breadth (IOB)	0.470	0.035	-0.368
Height of the Braincase (HB)	0.590	-0.223	-0.252
Length of mandible (LM)	0.820	-0.126	-0.045
% of total variance explained	49.876	11.087	9.110
Eigenvalue	6.484	1.441	1.184

Among the localities of our field surveys, *C. tanakae* was recorded at almost all sites investigated (Fig. 3), whereas *C. attenuata* was only found in the following six provinces: Sichuan Province (Baoxing), Fujian Province (Mount Wuyi), Hubei Province (Shennongjia), Guangdong Province (Nanling), Jiangxi Province (Mount Jinggang), and Zhejiang Province (Jinhua).

## Discussion

This study indicates that *C. attenuata* and *C. tanakae* are sympatrically distributed not only in continental Indochina (Jenkins et al. 2009, 2013; Bannikova et al. 2011; Abramov et al. 2012) but also in the mainland of China. The distribution of *C. attenuata* is apparently limited to only two ranges, i.e., Baoxing of Sichuan to Shennongjia of Hubei and Nanling of Guangdong to Jinhua of Zhejiang; the natural range of this species is much smaller than that of *C. tanakae* which is distributed almost all over the south of mainland China.

Note that the map of *C. attenuata* (Fig. 1, left) presented by the IUCN is erroneous due to the regular events of species misidentification of *C. tanakae* in the mainland of China. The IUCN map mistakenly shows the mixed distributions of both *C. attenuata* and *C. tanakae*; the presented distributions of *C. attenuata* in Taiwan and the Hainan Islands are erroneous for the same reason. For the distribution map of *C. tanakae* (Fig. 1, right), the range is not definitively established due to the few districts surveyed and information from more recent records has yet not to be included.

Based on morphological features we found among our samples and the results of its comparisons with type materials of *C. attenuata* and *C. tanakae* (Jenkins, et al., 2009, 2013), we consider that the specimens of the *C. attenuata* lineage should be attributed to *C. attenuata*, and the other lineage to *C. tanakae*. Wang (2003) divided *C. attenuata* into three subspecies in China, including the Himalayan subspecies (*C. a. rubricosa* Anderson, 1877) distributed in northwestern Yunnan (Gongshan), the South China subspecies (*C. a. attenuata* Milne-Edwards, 1872) distributed in other parts of mainland China and the Taiwan subspecies (*C. a. tanakae* Kuroda, 1938) distributed on Taiwan Island. It is clear that *C. a. tanakae* is actually a valid distinct species (Motokawa et al. 2001), but the other two subspecies still need taxonomical validation by detailed analysis to exclude the possibility of misidentification of *C. tanakae* specimens. Similarly, the same taxonomic challenge exists for *C. a. grisea* Howell, 1926, the subspecies distributed in the Fujian Province (Smith and Xie 2009). All these subspecies are uncertain because the authors may well have wrongly included specimens of *C. tanakae* mixed with *C. attenuata* samples.

There are many research reports listing *C. attenuata* in the mainland of China. For example, Zhang et al. (1987) investigated *C. attenuata* (*attenuate* in original paper) as a host animal of epidemic hemorrhagic fever, Wu (2002) reported population density fluctuation in *C. attenuata*, and many reports on animal diversity and pathogen host studies involved *C. attenuata*. Gu et al. (2007) reported that epidemiologic surveillance on leptospirosis in the Anhui Province and the first discovery of a pathogenic strain in the renal of *C. attenuata*, Wu et al. (2008) made a preliminary comparative anatomical study of digestive tracts between *C. attenuata* and *Apodemus agrarius*. Because *C. tanakae* might have been taxonomically misidentified with *C. attenuata* in these reports, and our present study demonstrates that *C. tanakae* is much more widely distributed in the mainland of China, the species “*C. attenuata*” described in these reports may be in fact *C. tanakae* or at least contains *C. tanakae*, results of these studies therefore need re-evaluation.

## Supplementary materials

Supplementary material 1 Tables S1–S3 Data type: species data Explanation note: **Table S1.** The information of *Crocidura tanakae* and *C. attenuata* specimens used in present study. **Table S2.** The specimen information of genus *Crocidura* and outgroup used in the present study. **Table S3.** Normality test of external and skull morphological indices of *C. attenuata* and *C. tanakae*. https://binary.pensoft.net/file/323541This dataset is made available under the Open Database License (http://opendatacommons.org/licenses/odbl/1.0/). The Open Database License (ODbL) is a license agreement intended to allow users to freely share, modify, and use this Dataset while maintaining this same freedom for others, provided that the original source and author(s) are credited.Yaoyao Li, Haotian Li, Masaharu Motokawa, Yi Wu, Masashi Harada, Huimei Sun, Xinmin Mo, Jing Wang, Yuchun Li

## References

[B1] Abramov AV Bannikova AA Rozhnov VV (2012) White-toothed shrews (Mammalia, Soricomorpha, *Crocidura*) of coastal islands of Vietnam. ZooKeys (207): 37–47. 10.3897/zookeys.207.3237 PMC3409683 22855639

[B2] Bannikova AA Lebedev VS Kramerov DA Zaitsev MV (2006) Phylogeny and systematics of the *Crocidura suaveolens* species group: corroboration and controversy between nuclear and mitochondrial DNA markers. Mammalia 70(1–2): 106–119. 10.1515/mamm.70.1-2.106

[B3] Bannikova A Abramov A Borisenko A Lebedev V Rozhnov V (2011) Mitochondrial diversity of the white-toothed shrews (Mammalia, Eulipotyphla, *Crocidura*) in Vietnam. Zootaxa 2812: 1–20. 10.11646/zootaxa.2812.1.1

[B4] Chen G Chen C Fu C Liu Y Chen S Yong B (2016) The complete mitogenome of Asian Gray Shrews, *Crocidura attenuata* (Soricidae). Mitochondrial DNA Part A 27(6): 3966–3967. 10.3109/19401736.2014.989507 25541314

[B5] Chen S Zhang Q Li F Wang X Wang Q Liu S (2018) A new record of *Crocidura tanakae* Kuroda, 1938 in Sichuan and Guizhou provinces. Acta Theriologica Sinica 38(2): 211–216.

[B6] Cheng F Wan T Chen Z Narayan P He K Jiang X (2017) First records of Taiwanese gray shrew (*Crocidura tanakae*) in Yunnan province, China. Chinese Journal of Zoology 52(5): 865–869.

[B7] Corbet G Hill J (1992) The mammals of the Indomalayan region: a systematic review. Oxford University Press, Oxford.

[B8] Ellerman J Morrison-Scott T (1951) Checklist of Palaearctic and Indian Mammals, 1758 to 1946: British museum. British Museum, Natural History.

[B9] Esselstyn J Timm R Brown R (2009) Do geological or climatic processes drive speciation in dynamic archipelagos? The tempo and mode of diversification in Southeast Asian shrews. Evolution 63(10): 2595–610. 10.1111/j.1558-5646.2009.00743.x 19500148

[B10] Esselstyn J Oliveros C (2010) Colonization of the Philippines from Taiwan: a multi-locus test of the biogeographic and phylogenetic relationships of isolated populations of shrews. Journal of Biogeography 37(8): 1504–1514. 10.1111/j.1365-2699.2010.02295.x

[B11] Fang Y Lee L Yew F Yu H (1997) Systematics of white-toothed shrews (*Crocidura*) (Mammalia: Insectivora: Soricidae) of Taiwan: Karyological and morphological Studies. Journal of Zoology 242(1): 151–166. 10.1111/j.1469-7998.1997.tb02936.x

[B12] Gu L Zhang Y Hu Y Wang J (2007) Epidemiologic surveillance on leptospirosis in Anhui Province and the first discovery of a pathogenic strain in the renal of *Crocidura attenuate*. Chinese Journal of Epidemiology 28(09): 929–930.

[B13] Hall TA (1999) BioEdit: a user-friendly biological sequence alignment editor and analysis program for Windows 95/98/NT. Nucleic Acids Symp Ser 41, 95–98.

[B14] Hutterer R﻿﻿ (1993) Order Insectivora In: Mammal Species of the World (2^nd^ edn). Smithsonian Institution Press, 69–130.

[B15] Irwin DM Kocher TD Wilson AC (1991) Evolution of the cytochrome b gene of mammals. Journal of Molecular Evolution 32(2): 128–144. 10.1007/BF02515385 1901092

[B16] Jameson E Jones G (1977) The Soricidae of Taiwan. Proceedings of the Biological Society of Washington 90(3): 459–482.

[B17] Jenkins PD Lunde DP Moncrieff CB (2009) Descriptions of new species of *Crocidura* (Soricomorpha: Soricidae) from mainland Southeast Asia, with synopses of previously described species and remarks on biogeography. Bulletin of the American Museum of Natural History 331: 356–405. 10.1206/582-10.1

[B18] Jenkins PD Abramov AV Bannikova AA Rozhnov VV (2013) Bones and genes: resolution problems in three Vietnamese species of *Crocidura* (Mammalia, Soricomorpha, Soricidae) and the description of an additional new species. Zookeys 313: 61–79. 10.3897/zookeys.313.4823 PMC3701231 23840165

[B19] Kuroda N (1938) A list of the Japanese mammals. Published by the author, Tokyo.

[B20] Lavrenchenko LA Bannikova AA Lebedev VS (2009) Shrews (*Crocidura* spp.) endemic to ethiopia: recent adaptive radiation of an ancient lineage. Doklady Biological Sciences 424(1), 57–60. 10.1134/S0012496609010177 19341086

[B21] Lei BY Yue Y Cui JF Ji SN Yu WH Han WB Zhou YB (2019) A new record of the Taiwanese gray shrew (*Crocidura tanakae* Kuroda, 1938) in Hubei Province. Acta Theriologica Sinica 39(02): 218–223. [in Chinese]

[B22] Li YC Lu HQ Zhang XD Xu WS (1989) Growth analysis and age indicator determination of striped hamster. Acta Theriologica Sinica 9(1): 49–55. [in Chinese]

[B23] Li YC Lu HQ Tian JX Hu JW (1990) Assessment of age indicators of greater long-tailed hamster by use of principal component analysis. Acta Theriologica Sinica 10(2): 121–127. [in Chinese]

[B24] Motokawa M Harada M Lin LG Koyasu K Hatteri S (1997) Karyological study of the gray shrew *Crocidura attenuate* (Mammalia: Insectivora) from Taiwan. Zoological Studies 36(1): 70–73.

[B25] Motokawa M Harada M Wu Y Lin LK Suzuki H (2001) Chromosomal polymorphism in the gray shrew *Crocidura attenuata* (Mammalia: Insectivora). Zoological Science 18(8): 1153–1160. 10.2108/zsj.18.1153

[B26] Motokawa M (2003) Geographic variation in the Japanese white-toothed shrew *Crocidura dsinezumi*. Acta Theriologica 48(2): 145–156. 10.1007/BF03194154

[B27] Ohdachi SD Iwasa MA Nesterenko VA Abe H Masuda R Haberl W (2004) Molecular phylogenetics of *Crocidura* shrews (Insectivora) in east and central Asia. Journal of Mammalogy 85(3): 396–403. 10.1644/1545-1542(2004)085<0396:MPOCSI>2.0.CO;2

[B28] Ohdachi S Iwasa M Vogel P Oshida T Lin L Abe H (2006) Molecular phylogenetics of soricid shrews (Mammalia) based on mitochondrial cytochrome *b* gene sequences: with special reference to the Soricinae. Journal of Zoology 270(1): 177–191.

[B29] Posada D (2008) jModelTest: Phylogenetic model averaging. Molecular Biology and Evolution 25: 1253–1256. 10.1093/molbev/msn083 18397919

[B30] Smith A Xie Y (2009) A guide to the mammals of China. Changsha: Hunan Education Publishing House. [Chinese version]

[B31] Tamura K Peterson D Peterson N Stecher G Nei M Kumar S (2011) MEGA5: Molecular evolutionary genetics analysis using maximum likelihood, evolutionary distance, and maximum parsimony methods. Molecular Biology and Evolution 28(10): 2731–2739. 10.1093/molbev/msr121 21546353 PMC3203626

[B32] Wang Y (2003) A complete checklist of mammal species and subspecies in China - a taxonomic and geographic reference. Beijing: China Forestry Publishing House. [in Chinese]

[B33] Wu A (2002) Population density fluctuation in *Crocidura attenuata*. Chinese Journal of Zoology 37(1): 60–61.

[B34] Wu Y Yuan XQ Hu JZ Yang Y Fang H Wang J (2008) Preliminary comparative anatomic study on digestive tract between *Crocidura attenuate* and *Apodemus agrarius*. Journal of China West Normal University (Natural Science) 29(1): 15–19.

[B35] Yang HF (1990) A brief comment on age determination methods for small mammals. Chinese Journal of Ecology 9(2): 54–55. [in Chinese]

[B36] Yang QS Xia L Ma Y Feng ZJ Quan GQ (2005) A Guide to the Measurement of Mammal Skull Ⅰ: Basic Measurement. Chinese Journal of Zoology 40(3): 50–56. 10.1360/982005-245 [in Chinese]

[B37] Yang QS Xia L Feng ZJ Ma Y Quan GQ Wu Y (2007) A Guide to the Measurement of Mammal Skull Ⅴ: Insectivora and Chiroptera. Chinese Journal of Zoology 42(2): 56–62. [in Chinese]

[B38] Zhang Y Zhao X Zhang B Shen J Tang J Bao M Wu G (1987) Investigation of *Crocidura attenuata* as a host animal with epidemic hemorrhagic fever (EHF). Chinese Journal of Public Health 6(4): 209–210. [in Chinese]

