# Supplementary material for: A revision of the geographical distributions of the shrews Crocidura tanakae and C. attenuata based on genetic species identification in the mainland of China
Source: Zookeys. 2019 Aug 5;869:147–60. doi: 10.3897/zookeys.869.33858 (PMC6690879; doi:10.3897/zookeys.869.33858)
Supplement: Supplementary material 1 [file zookeys-869-147-s001.docx]

**Supplementary**

Table S1. The information of *Crocidura tanakae* and *C. attenuata* specimens used in present study

| **Species** | **Genbank number** | **Field specimen code** | **Date** | **Collecting Locality** | **Longitude** | **Latitude** | **Altitude**  **(m)** | **Gender** |
| --- | --- | --- | --- | --- | --- | --- | --- | --- |
| *C. attenuata* | MK765684 | G09201 | 2009-09-22 | Nanling, Guangdong, China | 112.99 | 24.93 | 1831 | M |
|  | MK765685 | G09203 | 2009-09-22 | Nanling, Guangdong, China | 112.99 | 24.93 | 1831 | F |
|  | MK765708 | S1057 | 2014-07-30 | Wuyishan, Fujian, China | 117.66 | 27.74 | 1176 | M |
|  | MK765713 | S1414 | 2014-12-02 | Jinhua, Zhejiang, China | 119.64 | 29.12 | 56 | M |
|  | MK765714 | S1458 | 2014-12-06 | Jinhua, Zhejiang, China | 119.64 | 29.12 | 63 | M |
|  | MK765763 | S3358 | 2018-07-27 | Jinhua, Zhejiang, China | 119.63 | 29.20 | 572 | F |
|  | MK765768 | S3726 | 2018-08-23 | Jinggangshan, Jiangxi, China | 114.19 | 26.47 | 459 | F |
|  | MK765715 | S1610 | 2015-11-19 | Baoxing, Sichuan, China | 102.81 | 30.37 | 1144 | F |
|  | MK765716 | S1611 | 2015-11-19 | Baoxing, Sichuan, China | 102.81 | 30.37 | 1144 | F |
|  | MK765717 | S1615 | 2015-11-20 | Baoxing, Sichuan, China | 102.76 | 30.40 | 1145 | M |
|  | MK765718 | S1655 | 2015-11-21 | Baoxing, Sichuan, China | 102.75 | 30.40 | 1190 | F |
|  | MK765719 | S2014 | 2015-11-24 | Baoxing, Sichuan, China | 102.67 | 30.44 | 1525 | F |
|  | MK765720 | S2051 | 2015-11-24 | Baoxing, Sichuan, China | 102.68 | 30.46 | 1386 | F |
|  | MK765721 | S2216 | 2016-08-19 | Shennongjia, Hubei, China | 110.38 | 31.48 | 1225 | F |
|  | MK765722 | S2226 | 2016-08-19 | Shennongjia, Hubei, China | 110.39 | 31.48 | 1188 | F |
|  | MK765730 | S2537 | 2017-12-28 | Baoxing, Sichuan, China | 102.79 | 30.29 | 889 | M |
|  | MK765731 | S2540 | 2017-12-28 | Baoxing, Sichuan, China | 102.79 | 30.29 | 889 | M |
|  | MK765732 | S2565 | 2017-12-28 | Baoxing, Sichuan, China | 102.79 | 30.29 | 889 | M |
|  | MK765734 | S2567 | 2017-12-28 | Baoxing, Sichuan, China | 102.79 | 30.29 | 889 | F |
|  | MK765735 | S2576 | 2017-12-29 | Baoxing, Sichuan, China | 102.79 | 30.25 | 971 | M |
|  | MK765736 | S2577 | 2017-12-29 | Baoxing, Sichuan, China | 102.79 | 30.25 | 971 | M |
|  | MK765737 | S2670 | 2018-01-02 | Baoxing, Sichuan, China | 102.84 | 30.42 | 1078 | F |
|  | MK765738 | S2682 | 2018-01-02 | Baoxing, Sichuan, China | 102.84 | 30.42 | 1078 | M |
|  | MK765739 | S2717 | 2018-01-04 | Baoxing, Sichuan, China | 102.76 | 30.40 | 1215 | M |
|  | MK765740 | S2720 | 2018-01-04 | Baoxing, Sichuan, China | 102.76 | 30.40 | 1215 | F |
|  | MK765741 | S2767 | 2018-01-06 | Baoxing, Sichuan, China | 102.77 | 30.40 | 1032 | M |
|  | MK765742 | S2770 | 2018-01-06 | Baoxing, Sichuan, China | 102.77 | 30.40 | 1032 | M |
|  | MK765743 | S2771 | 2018-01-06 | Baoxing, Sichuan, China | 102.77 | 30.40 | 1032 | M |
|  | MK765744 | S2772 | 2018-01-06 | Baoxing, Sichuan, China | 102.77 | 30.40 | 1032 | F |
|  | MK765745 | S2798 | 2018-01-07 | Baoxing, Sichuan, China | 102.76 | 30.40 | 1128 | M |
|  | MK765746 | S2800 | 2018-01-07 | Baoxing, Sichuan, China | 102.76 | 30.40 | 1128 | M |
|  | MK765747 | S2801 | 2018-01-07 | Baoxing, Sichuan, China | 102.76 | 30.40 | 1128 | F |
|  | MK765748 | S2802 | 2018-01-07 | Baoxing, Sichuan, China | 102.76 | 30.40 | 1128 | F |
| *C. tanakae* | MK765695 | M04496 | 2004-09-26 | Emeishan, Sichuan, China | 103.48 | 29.61 | 900 | M |
|  | MK765733 | S2566 | 2017-12-28 | Baoxing, Sichuan, China | 102.79 | 30.29 | 889 | M |
|  | MK765749 | S2869 | 2018-01-09 | Yaan, Sichuan, China | 102.89 | 30.15 | 770 | M |
|  | MK765705 | MH8416 | 2002-09-24 | Emeishan, Sichuan, China | 103.32 | 29.55 | 2400 | F |
|  | MK765752 | S3155 | 2018-01-29 | Wuyishan, Fujian, China | 117.81 | 27.68 | 254 | M |
|  | MK765753 | S3185 | 2018-01-30 | Wuyishan, Fujian, China | 117.81 | 27.68 | 254 | M |
|  | MK765754 | S3186 | 2018-01-30 | Wuyishan, Fujian, China | 117.81 | 27.68 | 254 | F |
|  | MK765764 | S3508 | 2018-08-02 | Wuyishan, Fujian, China | 117.81 | 27.68 | 330 | F |
|  | MK765725 | S2468 | 2017-12-15 | Xiaogan, Hubei, China | 114.19 | 31.18 | 263 | F |
|  | MK765726 | S2475 | 2017-12-15 | Xiaogan, Hubei, China | 114.19 | 31.18 | 263 | M |
|  | MK765709 | S1370 | 2014-11-25 | Chongzuo, Guangxi, China | 106.96 | 22.47 | 271 | F |
|  | MK765710 | S1371 | 2014-11-25 | Chongzuo, Guangxi, China | 106.96 | 22.47 | 271 | F |
|  | MK765711 | S1385 | 2014-11-26 | Longzhou, Guangxi, China | 107.00 | 22.48 | 275 | M |
|  | MK765712 | S1404 | 2014-11-29 | Longzhou, Guangxi, China | 107.00 | 22.44 | 155 | M |
|  | MK765724 | S2438 | 2017-12-12 | Liuan, Anhui, China | 116.55 | 31.47 | 212 | F |
|  | MK765723 | S2426 | 2017-12-12 | Liuan, Anhui, China | 116.55 | 31.47 | 212 | M |
|  | MK765727 | S2477 | 2017-12-16 | Zhangjiajie, Hunan, China | 110.43 | 29.27 | 485 | F |
|  | MK765706 | S0703 | 2014-03-01 | Jishou, Hunan, China | 109.37 | 28.09 | 797 | F |
|  | MK765707 | S0731 | 2014-03-03 | Jishou, Hunan, China | 109.72 | 28.29 | 188 | F |
|  | MK765696 | M04505 | 2005-02-16 | Diaoluoshan, Hainan, China | 109.87 | 18.72 | 900 | M |
|  | MK765767 | S3654 | 2018-08-16 | Qiongzhong, Hainan, China | 109.80 | 19.09 | 323 | M |
|  | MK765728 | S2501 | 2017-12-21 | Peiling, Chongqing, China | 107.54 | 29.56 | 820 | M |
|  | MK765729 | S2523 | 2017-12-22 | Peiling, Chongqing, China | 107.54 | 29.56 | 820 | F |
|  | MK765750 | S2930 | 2018-01-17 | Qujing, Yunnan, China | 104.26 | 24.81 | 1538 | F |
|  | MK765771 | S3845 | 2018-08-29 | Shangrao, Jiangxi, China | 117.78 | 28.27 | 133 | M |
|  | MK765772 | S3846 | 2018-08-29 | Shangrao, Jiangxi, China | 117.78 | 28.27 | 133 | M |
|  | MK765773 | S3847 | 2018-08-29 | Shangrao, Jiangxi, China | 117.78 | 28.27 | 133 | F |
|  | MK765774 | S3850 | 2018-08-29 | Shangrao, Jiangxi, China | 117.78 | 28.27 | 133 | F |
|  | MK765775 | S3853 | 2018-08-29 | Shangrao, Jiangxi, China | 117.78 | 28.27 | 133 | F |
|  | MK765765 | S3552 | 2018-08-05 | Jinggangshan, Jiangxi, China | 114.22 | 26.47 | 318 | F |
|  | MK765769 | S3737 | 2018-08-23 | Jinggangshan, Jiangxi, China | 114.22 | 26.47 | 459 | F |
|  | MK765761 | S3772 | 2018-08-24 | Jinggangshan, Jiangxi, China | 114.22 | 26.47 | 359 | F |
|  | MK765760 | S3773 | 2018-08-24 | Jinggangshan, Jiangxi, China | 114.22 | 26.47 | 359 | M |
|  | MK765770 | S3774 | 2018-08-24 | Jinggangshan, Jiangxi, China | 114.22 | 26.47 | 359 | M |
|  | MK765755 | S3263 | 2018-08-24 | Shangrao, Jiangxi, China | 117.78 | 28.27 | 107 | F |
|  | MK765751 | S3114 | 2018-01-27 | Ningdu, Jiangxi, China | 115.97 | 26.54 | 314 | M |
|  | MK765756 | S3272 | 2018-02-03 | Jinhua, Zhejiang, China | 119.37 | 29.10 | 300 | M |
|  | MK765757 | S3273 | 2018-02-03 | Jinhua, Zhejiang, China | 119.37 | 29.10 | 300 | F |
|  | MK765776 | S3920 | 2018-08-31 | Jinhua, Zhejiang, China | 119.63 | 29.20 | 572 | M |
|  | MK765777 | S3921 | 2018-08-31 | Jinhua, Zhejiang, China | 119.63 | 29.20 | 572 | F |
|  | MK765778 | S3922 | 2018-08-31 | Jinhua, Zhejiang, China | 119.63 | 29.20 | 572 | M |
|  | MK765779 | S3954 | 2018-09-01 | Jinhua, Zhejiang, China | 119.63 | 29.20 | 109 | F |
|  | MK765780 | S3960 | 2018-09-01 | Jinhua, Zhejiang, China | 119.63 | 29.20 | 572 | F |
|  | MK765781 | S3984 | 2018-09-02 | Jinhua, Zhejiang, China | 119.63 | 29.20 | 109 | F |
|  | MK765782 | S3987 | 2018-09-02 | Jinhua, Zhejiang, China | 119.63 | 29.20 | 109 | M |
|  | MK765783 | S3988 | 2018-09-02 | Jinhua, Zhejiang, China | 119.63 | 29.20 | 109 | F |
|  | MK765784 | S4011 | 2018-09-03 | Jinhua, Zhejiang, China | 119.63 | 29.20 | 109 | M |
|  | MK765785 | S4012 | 2018-09-03 | Jinhua, Zhejiang, China | 119.63 | 29.20 | 109 | M |
|  | MK765786 | S4013 | 2018-09-03 | Jinhua, Zhejiang, China | 119.63 | 29.20 | 109 | M |
|  | MK765787 | S4038 | 2018-09-04 | Jinhua, Zhejiang, China | 119.63 | 29.20 | 109 | F |
|  | MK765788 | S4050 | 2018-09-04 | Jinhua, Zhejiang, China | 119.63 | 29.20 | 109 | F |
|  | MK765789 | S4060 | 2018-09-04 | Jinhua, Zhejiang, China | 119.63 | 29.20 | 109 | F |
|  | MK765790 | S4066 | 2018-09-04 | Jinhua, Zhejiang, China | 119.63 | 29.20 | 109 | M |
|  | MK765758 | S3318 | 2018-07-26 | Jinhua, Zhejiang, China | 119.62 | 29.20 | 542 | M |
|  | MK765759 | S3338 | 2018-07-26 | Jinhua, Zhejiang, China | 119.62 | 29.20 | 542 | F |
|  | MK765762 | S3345 | 2018-07-27 | Jinhua, Zhejiang, China | 119.63 | 29.20 | 572 | M |
|  | MK765682 | G09193 | 2009-09-21 | Nanling, Guangdong, China | 113.04 | 24.91 | 852 | M |
|  | MK765683 | G09196 | 2009-09-21 | Nanling, Guangdong, China | 112.99 | 24.93 | 1831 | M |
|  | MK765686 | G09266 | 2009-09-24 | Nanling, Guangdong, China | 113.08 | 24.89 | 820 | F |
|  | MK765687 | G09315 | 2009-09-29 | Yingde, Guangdong, China | 113.40 | 24.17 | 31 | M |
|  | MK765688 | G09316 | 2009-09-29 | Yingde, Guangdong, China | 113.40 | 24.17 | 31 | M |
|  | MK765689 | G09317 | 2009-09-29 | Yingde, Guangdong, China | 113.40 | 24.17 | 31 | F |
|  | MK765690 | G09320 | 2009-09-29 | Yingde, Guangdong, China | 113.40 | 24.17 | 31 | M |
|  | MK765692 | G12328 | 2012-08-19 | Shaoguan, Guangdong, China | 113.36 | 24.52 | 189 | F |
|  | MK765697 | MH8238 | 2001-09-30 | Yingde, Guangdong, China | 113.38 | 24.17 | 550 | F |
|  | MK765698 | MH8253 | 2001-10-01 | Yingde, Guangdong, China | 113.28 | 23.12 | 150 | M |
|  | MK765699 | MH8254 | 2001-10-01 | Yingde, Guangdong, China | 113.28 | 23.12 | 150 | F |
|  | MK765700 | MH8285 | 2001-10-03 | Longmen, Guangdong, China | 113.97 | 23.57 | 550 | M |
|  | MK765701 | MH8286 | 2001-10-03 | Longmen, Guangdong, China | 113.97 | 23.57 | 550 | M |
|  | MK765702 | MH8287 | 2001-10-03 | Longmen, Guangdong, China | 113.97 | 23.57 | 550 | F |
|  | MK765703 | MH8290 | 2001-10-04 | Guangzhou, Guangdong, China | 113.28 | 23.12 | 150 | M |
|  | MK765704 | MH8291 | 2001-10-04 | Guangzhou, Guangdong, China | 113.28 | 23.12 | 150 | M |
|  | MK765691 | G09325 | 2009-09-29 | Yingde, Guangdong, China | 113.40 | 24.17 | 31 | M |
|  | MK765766 | S3624 | 2018-08-10 | Yingde, Guangdong, China | 113.40 | 24.17 | 28 | F |
|  | MK765693 | G12350 | 2012-08-19 | Shaoguan, Guangdong, China | 113.36 | 24.52 | 189 | M |
|  | MK765694 | G12417 | 2012-08-21 | Shenzhen, Guangdong, China | 114.22 | 22.60 | 195 | M |
|  | MK765791 | MH8283 | 2001-10-03 | Longmen, Guangdong, China | 113.97 | 23.57 | 550 | F |

Table S2. The specimen information of genus *Crocidura* and outgroup used in the present study

| **Species** | **Genbank number** | **Locality** | **Longitude** | **Latitude** | **Literatures** |
| --- | --- | --- | --- | --- | --- |
| *C. tanakae* | AB175081 | Nantou Co, Taiwan, China | - | - | Ohdachi *et al*., 2006 |
|  | AB175080 | Nantou Co, Taiwan, China | - | - | Ohdachi *et al*., 2006 |
|  | GU358531 | Taiwan, China | 121°10' | 25°10' | Esselstyn and Oliveros,2010 |
|  | KX946002 | Guizhou, China | 110°33' | 31°14' | Chen *et al*., 2018 |
|  | KX946003 | Guizhou, China | 110°59' | 31°12' | Chen *et al*., 2018 |
|  | HM587017 | Lam Dong Province, Vietnam | 108°41' | 12°11' | Bannikova *et al*., 2011 |
|  | HM587022 | Lam Dong Province, Vietnam | 108°48' | 12°11' | Bannikova *et al*., 2011 |
|  | HM587027 | Binh Phuoc Province, Vietnam | 107°12' | 12°11' | Bannikova *et al*., 2011 |
|  | HM587028 | Binh Phuoc Province, Vietnam | 107°12' | 12°11' | Bannikova *et al*., 2011 |
|  | HM587031 | Khammouane Province, Laos | 104°49' | 17°33' | Bannikova *et al*., 2011 |
|  | HM587032 | Khammouane Province, Laos | 104°49' | 17°33' | Bannikova *et al*., 2011 |
|  | EU122211 | Vinh Phu Province, Vietnam | 105°38' | 21°27' | Bannikova *et al*., 2011 |
| *C. attenuata* | JX181934 | Cat Ba Island, Vietnam | 106°56' | 20°48' | Abramov *et al*., 2012 |
|  | JX181935 | Cat Ba Island, Vietnam | 106°56' | 20°48' | Abramov *et al*., 2012 |
|  | AB175082 | Ha Giang Province, Vietnam | 104°50' | 22°45' | Ohdachi *et al*., 2006 |
|  | AB175083 | Ha Giang Province, Vietnam | 104°50' | 22°45' | Ohdachi *et al*., 2007 |
|  | KP120863 | Ganluo, Sichuan, China | 102°53' | 29°13' | Chen *et al*., 2016 |
|  | NC026204 | Ganluo, Sichuan, China | 102°53' | 29°13' | Chen *et al*., 2016 |
| *C. suaveolens* | AB077280 | Wien, Austria | - | - | Ohdachi *et al*., 2004 |
| *C. gueldenstaedtii* | AY994373 | Tuapse, Russia | - | - | Bannikova *et al*., 2006 |
| *C. sibirica* | AB077087 | Mosuowan, Xinjiang, China | - | - | Ohdachi *et al*., 2004 |
| *C. macmillani* | EU742601 | Ethiopia | - | - | Bannikova *et al*., 2009 |
| *Suncus murinus* | NC024604 | Yulin, Guangxi, China | - | - | Chen *et al*., 2016 |

Table S3 Normality test of external and skull morphological indices of *C. attenuata* and *C. tanakae*

|  | *C. attenuata* | | | | | *C. tanakae* | | | | |
| --- | --- | --- | --- | --- | --- | --- | --- | --- | --- | --- |
|  | n | Mean | SD | Z | P | n | Mean | SD | Z | P |
| Total Body Length (TBL) | 26 | 133.92 | 6.22 | 0.139 | 0.200 | 64 | 132.95 | 6.84 | 0.115 | **0.036** |
| Head and Body Length (HBL) | 26 | 77.96 | 3.54 | 0.107 | 0.200 | 64 | 78.90 | 5.42 | 0.118 | **0.028** |
| Ear Length (EL) | 26 | 8.32 | 1.05 | 0.166 | 0.065 | 63 | 8.79 | 1.00 | 0.116 | **0.036** |
| Greatest Length of Skull (GLS) | 23 | 20.99 | 0.59 | 0.152 | 0.182 | 61 | 20.54 | 0.61 | 0.082 | 0.200 |
| Cranial base Length (GBL) | 24 | 20.91 | 0.57 | 0.137 | 0.200 | 61 | 20.39 | 0.61 | 0.082 | 0.200 |
| Median palatal Length (MPL) | 26 | 9.07 | 0.41 | 0.153 | 0.120 | 63 | 8.89 | 0.31 | 0.072 | 0.200 |
| Length of tooth row (LUTR) | 26 | 9.36 | 0.26 | 0.102 | 0.200 | 63 | 9.16 | 0.34 | 0.082 | 0.200 |
| Greatest palatal breadth (GPB) | 26 | 6.25 | 0.16 | 0.131 | 0.200 | 64 | 6.32 | 0.31 | 0.119 | **0.026** |
| Breadth of Occipital Condyles (BOC) | 22 | 5.20 | 0.19 | 0.085 | 0.200 | 60 | 5.13 | 0.23 | 0.135 | **0.008** |
| Greatest Breath of Braincase (BBC) | 26 | 9.59 | 0.27 | 0.123 | 0.200 | 64 | 9.26 | 0.32 | 0.060 | 0.200 |
| Interorbital Breadth (IOB) | 26 | 3.97 | 0.23 | 0.171 | 0.050 | 64 | 3.79 | 0.17 | 0.099 | 0.192 |
| Height of the Braincase (HB) | 26 | 5.13 | 0.14 | 0.148 | 0.150 | 64 | 5.01 | 0.15 | 0.094 | 0.200 |
| Length of mandible (LM) | 26 | 10.01 | 0.28 | 0.140 | 0.200 | 64 | 9.85 | 0.33 | 0.074 | 0.200 |
